# Evaluation of the Active Melioidosis Detect™ test as a point-of-care tool for the early diagnosis of melioidosis: a comparison with culture in Laos

**DOI:** 10.1093/trstmh/trz092

**Published:** 2019-10-22

**Authors:** Maria Chiara Rizzi, Sayaphet Rattanavong, Latsaniphone Bouthasavong, Amphayvanh Seubsanith, Manivanh Vongsouvath, Viengmon Davong, Annalisa De Silvestri, Tommaso Manciulli, Paul N Newton, David A B Dance

**Affiliations:** 1 University of Pavia, Pavia, Italy; 2 Lao-Oxford-Mahosot Hospital-Wellcome Trust Research Unit (LOMWRU), Microbiology Laboratory, Mahosot Hospital, Vientiane, Lao PDR; 3 Centre for Tropical Medicine and Global Health, University of Oxford, Oxford OX3 7FZ, UK; 4 Faculty of Infectious and Tropical Diseases, London School of Hygiene and Tropical Medicine, London WC1E 7HT, UK

**Keywords:** *Burkholderia pseudomallei*, immunoassay, Laos, melioidosis, point-of-care technology

## Abstract

**Background:**

Melioidosis is difficult to diagnose clinically and culture of *Burkholderia pseudomallei* is the current, imperfect gold standard. However, a reliable point-of-care test (POCT) could enable earlier treatment and improve outcomes.

**Methods:**

We evaluated the sensitivity and specificity of the Active Melioidosis Detect™ (AMD) rapid test as a POCT and determined how much it reduced the time to diagnosis compared with culture.

**Results:**

We tested 106 whole blood, plasma and buffy coat samples, 96 urine, 28 sputum and 20 pus samples from 112 patients, of whom 26 (23.2%) were culture-positive for *B. pseudomallei*. AMD sensitivity and specificity were 65.4 and 87.2%, respectively, the latter related to 10 weak positive reactions on urine samples, considered likely false positives. The positive predictive value was 60.7%, negative predictive value was 89.3% and concordance rate between operators reading the test was 95.7%; time to diagnosis decreased by a median of 23 h.

**Conclusions:**

Our findings confirm that a strongly positive AMD result can reduce the time to diagnosis of melioidosis. However, the AMD currently has a disappointing overall sensitivity, especially with blood fractions, and specificity problems when testing urine samples.

## Introduction

Melioidosis is an infectious disease caused by *Burkholderia pseudomallei*, a gram-negative, oxidase-positive, saprophytic environmental bacillus.^1^ The infection is highly endemic in South-East Asia and northern Australia,^2^ but is also widely underreported.^3^ The incidence is highest during the rainy season (May–October in South-East Asia), especially following severe weather events.^4–6^ The mortality rate ranges from 20 to 50%, rendering melioidosis a common cause of death in some areas.^7^ In low-resource settings, the wide differential diagnosis can prove problematic for physicians, and has led to the nickname ‘the great mimicker’.^8^

Currently, the gold standard for diagnosis is culture, using selective media such as Ashdown's agar and selective broth for sites with a normal flora. However, in settings such as Laos, these are rarely available.^9^ Furthermore, the bacterium can be easily misidentified.^9^ Moreover, the culture methods currently used have a sensitivity that may be as low as 60.2%,^10^ and take several days before the diagnosis is confirmed,^11^ leading to potentially fatal delays before the patient receives appropriate treatment.^12^ New tools are therefore needed to diagnose the disease rapidly and accurately. Recently, a qualitative, membrane-based lateral flow immunoassay point-of-care test (POCT) that detects the capsular polysaccharide of *B. pseudomallei*, the Active Melioidosis Detect™ (AMD), has been developed by InBios (Seattle, WA, USA).^13^ The AMD has undergone a small number of evaluations for the direct detection of *B. pseudomallei* in a range of clinical samples^12–17^ as well as for the detection of the organism in blood culture broths.^15^^,^^18^ Such a test, which is easy to use and relatively cheap, could prove a very valuable tool in resource-limited settings.

In this study, we aimed to evaluate the AMD as a POCT for suspected melioidosis, testing all available samples in real time as soon as possible after the admission of the patient, to compare its sensitivity, specificity and time to presumptive diagnosis with that of culture.

## Methods

### Study site and population

The study was carried out in Mahosot Hospital, Vientiane, Lao People's Democratic Republic. The hospital serves patients from Vientiane and is also a main national referral hospital for patients from other provinces. The number of patients with melioidosis diagnosed in the hospital has been steadily increasing in recent years.^19^ Since melioidosis is highly protean in its manifestations, study entry was simply based on a clinical suspicion of melioidosis by the responsible local physician. Patients were actively recruited by one of the investigators (MCR) visiting the Adult and Pediatric Infectious Diseases, Ear-Nose-Throat, General Medicine, Adult Intensive Care Unit, Pulmonary and Gastroenterology/Hematology wards at Mahosot Hospital at least once a day. The study was conducted throughout the rainy season (from June to October) in 2017.

### Patient enrolment and sampling

All patients with clinically suspected melioidosis were considered for inclusion. The patient or a legal representative was asked to provide written informed consent before being enrolled. A standard set of samples was obtained as soon as possible after melioidosis was suspected by the local physician. This included blood cultures, EDTA blood, throat swab, urine, sputum, pus and body fluids (e.g. pleural or joint effusion) when clinically indicated and feasible. All patients from whom *B. pseudomallei* was isolated were started on the standard regimen^20^ for the treatment of melioidosis as soon as possible after the laboratory informed the responsible clinician of the suspected diagnosis. When an AMD was positive, the result was communicated by a member of the laboratory clinical team to the physician caring for the patient, explaining that the test was under evaluation, and a decision was made about the need for treatment based on the test result in the context of the clinical and epidemiological features. Treatment and outcome of each case were recorded on a standard proforma.

### Laboratory procedures

All samples obtained were tested as soon as possible after their receipt in the laboratory. The AMDs were performed according to the manufacturer’s instructions (see the Supplementary Data) for all samples except blood. For these, an aliquot of the EDTA blood sample was taken and the remaining EDTA blood was separated into plasma and buffy coat fractions by centrifugation (2060 rpm for 8 min). Next, 35 μl of each fraction was added to one drop of lysis buffer and then 35 μl of the mixture was added to three drops of chase buffer, mixed with a pipette and tested.

The results were read independently after 15 min by two different operators, one of whom was blind to clinical details. Line intensity was defined as ‘strong’ if the line was clearly visible to the naked eye and in a photograph, and ‘weak’ if the line was hard to see with the naked eye and/or in a photograph. When discordant results were obtained, the AMD strips were reviewed by a third person who decided the line intensity. Weak lines were considered positive only if both investigators agreed that they were positive.

Blood cultures were processed as described:^21^ the bottles were incubated in air at 37°C for 7 d, examined daily and, if turbid, were subcultured onto blood agar, plus chocolate and MacConkey agar if gram-negative rods were observed on the gram stain of the broth. Pus and sputum samples were cultured directly on non-selective media, Ashdown agar and in enrichment broths as described.^11^ Centrifuged deposits of urine were cultured on Ashdown agar. Any gram-negative, oxidase-positive rods and all blood culture broths containing gram-negative rods were tested with a latex agglutination reagent specific for the extracellular polysaccharide of *B. pseudomallei*.^22^ Confirmation of identity of latex-positive colonies was performed by testing with API20NE (bioMérieux, Basingstoke, UK). The time between the receipt of samples until the first positive AMD result and the first positive latex agglutination test was recorded.

### Statistical analysis

We predicted the recruitment of 40 patients based on data from previous rainy seasons. Assuming a sensitivity of the AMD of 75%, the precision of the sensitivity with this sample size was estimated as being between 59 and 87% (95% CIs), which would be similar to or higher than the reported sensitivity of culture (60.2%).^10^ Data analysis was performed with STATA statistical package release 14.0 (Stata Corporation, College Station, TX, USA) and MedCalc (https://www.medcalc.org/calc/diagnostic_test.php; Ostend, Belgium). We performed a Shapiro-Wilk analysis to test the normal distribution of quantitative variables. When quantitative variables were normally distributed, the results were expressed as the mean with 95% CIs, otherwise median and IQR (25th–75th percentile) were reported. Qualitative variables were summarized as counts and percentages to calculate sensitivities, specificities, the positive predictive value (PPV), the negative predictive value (NPV) and the estimated 95% CIs. Results were initially analyzed by patient according to whether or not they had any positive culture for *B. pseudomallei*. Concordance between the AMD and culture were evaluated with Cohen’s kappa; the kappa-statistic measure of agreement varies from 0 (no match) to 100 (perfect match). Analysis was also performed by specimen type: AMD results on blood fractions were compared with concurrently taken blood cultures, otherwise AMD was compared with culture results on the same specimen. The Landis and Koch criteria^23^ were used to categorize agreement between the two operators as follows: 0: poor; 0–20: slight; 21–40: fair; 41–60: moderate; 61–80: substantial; 81–100: almost perfect. To assess statistical significance of the difference between time to diagnosis with AMD and culture, we used a Wilcoxon signed-rank test. A p-value of <0.05 was considered significant.

## Results

### Patients and samples

We enrolled 112 patients with suspected melioidosis during the study period, of whom 59 were female (52.7%). Twenty-six patients (23%), including 12 females (46.2%), proved to have culture-positive melioidosis. From the 112 patients, we collected: 106 whole blood plasma and buffy coat samples, all of which had concurrent blood cultures; 96 urine samples (including 86 midstream and 10 catheter urine samples); 28 sputum samples and 20 pus samples. In addition, 107 throat swabs from these patients were cultured for *B. pseudomallei*, which is part of the normal local diagnostic work-up for patients with suspected melioidosis.^24^ Throat swabs and blood cultures were not available for five and six patients, respectively, as these samples were not requested by the physician responsible. Details of the patients and their ward locations are shown in Table 1.

**Table 1:** Demographic features and locations of patients

Class	All patients recruited (n=112)	*Burkholderia pseudomallei* culture-positive patients (n=26)	*Burkholderia pseudomallei* AMD-positive patients (n=28)
Age, y			
<15	11 (9.8%)	5 (19.3%)	4 (14.3%)
15–39	22 (19.6%)	5 (19.3%)	5 (17.9%)
40–60	51 (55.5%)	11 (42.3%)	12 (42.9%)
>60	28 (25%)	5 (19.3%)	7 (25%)
Gender			
Female	59 (52.7%)	12 (46.1%)	11 (39.3%)
Male	53 (47.3%)	14 (53.8%)	17 (60.7%)
Ward of recruitment			
General Medicine	25 (22.3%)	1 (3.8%)	5 (17.9%)
Pulmonary	20 (19.6%)	5 (19.3%)	6 (21.4%)
Adult Intensive Care Unit	18 (16.1%)	6 (23.1%)	3 (10.7%)
Ear-Nose-Throat	16 (14.3%)	5 (19.3%)	4 (14.3%)
Adult Infectious Diseases	15 (13.4%)	6 (23.1%)	6 (21.4%)
Gastroenterology and Hematology	8 (7.1%)	0	1 (3.6%)
Pediatric Infectious Diseases	7 (6.2%)	1 (3.8%)	1 (3.6%)
Surgery	2 (1.8%)	2 (7.7%)	2 (7.1%)
International Clinic	1 (0.9%)	0	0

Of the 26 patients with culture-positive melioidosis, 22 (84.2%) received specific treatment for melioidosis during hospital admission. Among the latter, 18 survived (81.8%) and four died (18.2%). Three of the 26 culture-positive cases (12%) did not receive specific treatment for melioidosis (one refused treatment and two died before they could be treated) and one (3.8%), who had originally been admitted to Mahosot Hospital, was subsequently transferred and treated elsewhere and information about their management and outcome was not available.

### Analysis by patient

In total, 26 patients had melioidosis confirmed by culture, of whom 17 (65.4%) had positive AMD results from at least one sample. Of the 86 patients with negative culture, 75 (87.2%) were negative by AMD on all available samples. The AMD thus showed a sensitivity of 65.4% (95% CI 44.3 to 82.8%), a specificity of 87.2% (95% CI 78.3 to 93.4%), a PPV of 60.7% (95% CI 45.4 to 74.2%) and a NPV of 89.3% (95% CI 83 to 93.4%) for the detection of culture-positive melioidosis in the ‘by patient’ analysis. The concordance rate between AMD and culture was 54.3% (95% CI 36.2 to 72.3%).

### Analysis by sample type

The agreement between the two operators reading the AMD results was 95.7% (95% CI 90.8 to 100%), corresponding to an almost perfect match following Landis and Koch criteria.^23^ The results were analyzed by sample type, using culture as the gold standard, as shown in Table 2. Sensitivities ranged from 0% (95% CI 0 to 26.5%) for buffy coat to 85.7% (95% CI 42.1 to 99.6%) for pus. Specificities ranged from 79.6% (95% CI 70 to 87.2%) for urine to 100% for whole blood, buffy coat, sputum and pus. PPVs ranged from 9.5% (95% CI 4.1 to 20.5%) for urine to 100% for whole blood, sputum and pus, and NPVs ranged from 88.7% (95% CI 88.7 to 88.9%) for buffy coat to 98.7% (95% CI 93.7 to 99.7%) for urine.

**Table 2:** Culture-Active Melioidosis *Detect*™ (AMD) comparison by sample type

Sample type	No.	Culture-positive	AMD- positive	Possible AMD ‘false positives’	Sensitivity (%) (95% CI)	Specificity (%) (95% CI)	PPV (%) (95% CI)	NPV (%) (95% CI)
Whole blood	106	12^a^	2	0	16.7 (2.9 to 48.4)	100 (96.2 to 100)	100	90.4 (88 to 92.4)
Plasma	106	12^a^	4	1	25 (5.5 to 57.2)	98.9 (94.2 to 100)	75 (25.3 to 96.4)	91.2 (88.2 to 93.5)
Buffy coat	106	12^a^	0	0	0 (0 to 26.5)	100 (96.2 to 100)	-	88.7 (88.7 to 88.9)
Urine	96	3	21	19	66.7 (9.4 to 99.2)	79.6 (70 to 87.2)	9.5 (4.1 to 20.5)	98.7 (93.7 to 99.7)
Sputum	28	5	4	0	80 (28.4 to 99.5)	100 (85.2 to 100)	100	95.8 (79.9 to 99.3)
Pus	20	7	6	0	85.7 (42.1 to 99.6)	100 (75.3 to 100)	100	92.9 (67.3 to 98.7)

^a^For blood samples the comparison was made between the type of sample listed in the table for AMD and a simultaneously collected blood culture. NPV, negative predictive value; PPV, positive predictive value.

### Positive urine results

Three patients had melioidosis confirmed by urine culture. Of these, two (66.7%) had a positive urine AMD result. Of the 93 patients with a negative urine culture, 19 (20%) had a positive urine AMD result. However, nine (42.9%) of these 19 ‘false positives’ came from patients who grew *B. pseudomallei* from other samples (including throat swabs) and were thus possibly detecting genuine antigenuria, whereas 10 (47.6%) were culture-negative on all samples obtained, resulting in an overall sensitivity for a positive urine AMD in detecting culture-positive melioidosis of 66.7% (95% CI 9.4 to 99.2%) and a specificity of 79.6% (95% CI 70 to 87.2%). Of the nine positive AMDs from patients with positive cultures at other sites, eight were read as positive by both operators (three as strong lines and five as weak lines), while one was read as positive only by the operator totally blind to clinical details but was not confirmed by the third operator. The latter patient had a deep pus sample that was positive by both culture and AMD. Of the other eight patients, seven had positive cultures and/or AMD from more than one site, whereas one was culture-positive only from a throat swab.^24^

All 10 patients whose urine AMDs were positive but had no evidence of culture-positive melioidosis gave only weak lines in the AMD test. Eight of these were considered likely to be genuinely false-positive reactions, as the clinical presentations and courses of the patients were not consistent with melioidosis since they were discharged in good condition despite having been treated with antibiotics that are not effective in melioidosis. The interpretation in the other two patients is unclear: one started IV ceftazidime but was discharged for treatment elsewhere and one was lost to follow-up, so their outcomes are unknown.

These results are summarized in Table 3.

**Table 3:** Urine active melioidosis detect (AMD) results in patients with culture-positive melioidosis in sites other than urine

Patient code	AMD strength on urine	Other positive AMD	*Burkholderia pseudomallei* culture positive samples
011	Strong line	-	Blood culture, sputum
035	Weak line	Sputum	Throat swab, sputum
048	Weak line^a^	Deep pus	Deep pus (back abscess)
055	Weak line	EDTA plasma	Blood culture, throat swab
067	Weak line	Throat swab sputum	Blood culture
		EDTA whole blood	Throat swab
		EDTA plasma	Sputum
078	Strong line	-	Blood culture, pus swab (leg lesion)
086	Strong line	Deep pus EDTA plasma	Deep pus (spleen abscess)
092	Weak line	EDTA whole blood	Blood culture
		EDTA plasma	Pus swab (lumbar abscess)
105	Weak line	-	Throat swab

^a^This result was read as positive by only one of the two operators and was not confirmed by the third operator, so was read as negative.

### Time to diagnosis

In 16 (61.5%) culture-positive patients, a positive AMD result was available earlier than culture, but only in five did this actually reduce the time until the patient received appropriate treatment, as in six cases clinicians decided to wait for culture results, in three cases treatment for melioidosis had already been given on the basis of clinical suspicion and in two cases no melioidosis-specific treatment was given because one patient died too early and the other declined treatment. The time from sample collection to the first positive AMD result or presumptive positive culture result for those patients positive in both tests was reduced by a median of 23.1 h by the AMD (IQR 6 h, p=0.003).

## Discussion

Infection with *B. pseudomallei* is frequently severe, with septic shock occurring in up to 21% of patients.^25^ The early administration of specific antibiotic treatment is key for securing a favorable outcome. A simple, sensitive and specific rapid diagnostic test that can be performed directly on clinical samples would be highly valuable, particularly as melioidosis is common in areas where laboratory facilities and training are often scarce. Culture, PCR and immunofluorescence assays^26^^,^^27^ often cannot be routinely used in melioidosis-endemic areas because they require dedicated facilities and highly trained staff. Tests for antibodies to *B. pseudomallei* using techniques such as indirect haemagglutination, while widely used, are beset with problems of both sensitivity and specificity,^28^ although assays using better characterized antigens are being developed and show more promise as diagnostic tools.^29^ Latex agglutination is useful for rapid testing of blood cultures growing gram-negative bacilli, but requires an initial incubation step, which inevitably delays the time to positive results.^30^ The clinical management of melioidosis could thus be improved by a POC test. This study built on our previous evaluation of the AMD, which was conducted at the same hospital,^15^ but instead of using specimens passively received in the laboratory, we actively encouraged the submission of all relevant specimens (blood and urine, and sputum and pus whenever available) from suspected melioidosis patients as soon as possible after hospital admission. Using this approach we found that for patients with a positive culture, the time to presumptive diagnosis was reduced by a median of 23 h (p=0.003). In five cases, this actually led to a reduction in the time between admission and the start of treatment, and this might have happened in more patients had the test been fully evaluated and licensed. However, two of these five patients died, while three survived. A much larger study would be needed to determine whether this test actually has the potential to reduce mortality in patients with melioidosis. In practical terms, the AMD is user-friendly as it requires little training and can give results in only 15 min. Moreover, in our hands the inter-operator concordance was good. Although the test is not yet available commercially, if marketed at relatively low cost it could be suitable for resource-limited melioidosis-endemic settings where culture facilities are frequently not available.

Nonetheless, there is clearly room for improvement, as our ‘by patient’ performance analysis of the AMD showed an overall disappointing sensitivity (65.4%) and specificity (87.2%) for detecting culture-positive melioidosis. These are lower than the 85.7% sensitivity and 93.6% specificity by sample reported by Shaw et al.^14^ The low AMD specificity in our study was almost entirely due to weak positive reactions obtained on urine samples and read as positive by at least one operator strictly following the manufacturer’s instructions. On clinical grounds, we believe the majority of these to be false positives, the cause of which requires further investigation. Similar problems with urine samples were also reported by Shaw et al.^14^ In our ‘by sample’ analysis, the AMD performed directly on the various blood fractions had a very low sensitivity compared with blood cultures (0% for buffy coat, 16.7% for whole blood and 25% for plasma), as previously reported by Robertson et al.^12^ but so far not analyzed in other studies. This presumably relates to low levels of circulating antigen, even in patients with bacteremia and the comparatively large volumes of blood that are cultured. Sputum and pus samples showed good sensitivities in our study (80 and 85.7%, respectively), which were higher than the results obtained by Woods et al. (sputum sensitivity 33.3%, pus 47.1%)^15^ and closer to the sensitivity for pus samples found by Shaw et al. (93.1%).^14^ However, these two sample types were only available in 28 (25%) and 20 (22.4%) of the patients recruited, and seven (25%) and five (25%) of the 26 culture-positive patients, respectively. Thus, our results suggest that the collection of these samples in patients suspected of having melioidosis is of great importance wherever possible.

One possible explanation for false negative cultures is prior antibiotic treatment, which is common in patients admitted to hospital in Laos*.*^31^ Patients are routinely questioned about this by medical staff although they are often unaware of the agents they have taken or even whether these were antibiotics. However, we feel that this is unlikely to have had a significant impact in our study as the oral agents readily available in Laos, such as ampicillin and amoxicillin, have little or no activity against *B. pseudomallei*, and even in hospitals the agent most commonly used for empirical treatment of sepsis, ceftriaxone, has low clinical efficacy in melioidosis.^32^

The polysaccharide antigen detected by the AMD has been shown to be largely eliminated by renal filtration in a murine model, forming a single-walled nanotube structure that may allow its filtration through the glomerulus despite its high molecular weight of 300 kDa.^33^ Thus, even when viable *B. pseudomallei* are not present in urine, antigenuria might result in a positive AMD. Urinary antigen detection appeared promising as a diagnostic test for melioidosis in our previous evaluation of the AMD^15^ with a sensitivity of 86.7% compared with culture.^15^ Urine AMD had a lower sensitivity in the current evaluation (66.7%), although again we found evidence of probable antigenuria in nine patients who had a negative urine culture but a positive culture from other sites. However, in this evaluation we found problems with specificity of urine antigen detection. One possible explanation for this is that the methodology for testing urine in this study, as specified in the latest manufacturer’s instructions, differed from that in our previous study, in which neat urine was tested. This requires further evaluation. We believe that the majority of these weak bands were likely to have been false positives as discussed above, but in future the use of an automated reader to quantify line intensity or urine concentration may help to distinguish between genuine antigenuria and false positive reactions.

## Conclusions

Our study has confirmed the potential for a POC test to enable earlier, potentially life-saving treatment in patients with melioidosis. Sensitivity and specificity are good with pus and sputum if these can be obtained, and a strong positive band on the AMD, as currently formulated, is sufficient evidence to start a patient on treatment for melioidosis. Urine antigen detection is promising, but further developments such as the modification of the test characteristics, implementation of an automated reader or the use of urine concentrators may help to distinguish between true and false positives. However, evaluations of the AMD undertaken to date do not suggest that the AMD is likely to be useful for testing blood samples. It should also be noted that, as with all diagnostic tests, the sensitivity and specificity will never be 100% and so some patients will need to be managed on the basis of a strong clinical suspicion of melioidosis.

## Supplementary Material

SupplementaryData_trz092
