# Supplementary material for: Evaluation of the Active Melioidosis Detect™ test as a point-of-care tool for the early diagnosis of melioidosis: a comparison with culture in Laos
Source: Trans R Soc Trop Med Hyg. 2019 Oct 22;113(12):757–63. doi: 10.1093/trstmh/trz092 (PMC6907004; doi:10.1093/trstmh/trz092)
Supplement: SupplementaryData_trz092 [file supplementarydata_trz092.pdf]

# Active Melioidosis *Detect* ™ Rapid Test

**Rapid Detection of the capsular polysaccharide (CPS) produced by *Burkholderia pseudomallei***

***For Research Use Only - Not for use in diagnostic procedures***

## INTENDED USE

The Active Melioidosis *Detect*™ (AMD) point of care diagnostic test, for the detection of *Burkholderia pseudomallei* (the causative agent of melioidosis) is a sensitive rapid immunochromatographic strip assay for the qualitative detection of capsular polysaccharide (CPS) produced by *B. pseudomallei* and *B. mallei* (1). The AMD can be used to test: i) human blood from a finger stick or ii) serum or plasma obtained from human blood iii) other samples e.g. urine, pus, sputum and bacterial colonies or hemoculture bottles.

**For use in laboratories with experienced personnel who have training in principles and use of microbiological culture identification methods, appropriate biosafety equipment and containment. For research use only. Not for use in diagnostic procedures.**

***This assay is not FDA cleared or approved for testing blood or plasma donors.***

***Warning:*** *The performance of the assay has not been fully established. Assay interference from other factors such as high level of lipid, protein, HAMA, bilirubin, are yet to be established.*

## SUMMARY AND EXPLANATION OF THE TEST

Melioidosis is a devastating disease caused by infection with the bacterium *B. pseudomallei*. A wide range of clinical manifestations develop during melioidosis. Patient presentation can vary from acute fulminant septic illness to chronic disease (2). The incidence of melioidosis is highest in the endemic regions of Thailand and Australia. A recent prospective study has determined that melioidosis incidence rates have been increasing in northeast Thailand from 1997-2006 and the mortality rate during this period was nearly 43% (3). In the same geographical region, melioidosis is the third most common cause of death from infectious disease after HIV/AIDS and tuberculosis (3). In regions of northern Australia, where intensive care treatment is more readily available, the mortality rate is still unacceptably high at around 10% (4). Relapse rates can approach 25%, due to the intracellular survival of *B. pseudomallei* (5, 6).

Detection of *B. pseudomallei* by diagnostic microbiology laboratories is difficult due to limited experience of personnel and a lack of validated diagnostic reagents (7). In addition, the clinical manifestations of melioidosis mimic those of common acute and chronic bacterial infections, further complicating diagnosis. Isolation of *B. pseudomallei* from cultures of patient samples still remains the "gold standard" for the diagnosis of melioidosis (2). Unfortunately, culturing for *B. pseudomallei* is labor intensive; samples of blood, urine, pus, and sputum must be collected. Isolation of *B. pseudomallei* from any one of these cultures is diagnostic for melioidosis (8, 9), however, this takes 3-7 days (10). This time frame is not favorable for administration of the proper antibiotics effective against *B. pseudomallei*. In addition, *B. pseudomallei* may be dismissed as a culture contaminant or be misidentified by standard identification methods or automated bacterial identification systems. **Therefore, the Active Melioidosis *Detect*™ is being developed to provide a rapid and accurate diagnosis in order to improve patient outcome.**

## PRINCIPLE

The Active Melioidosis *Detect*™ Rapid Test is a qualitative, membrane-based immunoassay for the detection of CPS produced by *B. pseudomallei* during infection. The membrane is pre-coated with a specific monoclonal antibody to CPS on the test line region and a control antigen on the control line region. During testing, the sample reacts with the colloidal gold conjugate which has been pre-coated in the test device. The mixture then migrates upward on the membrane chromatographically and complexes with CPS from the sample and monoclonal antibody on the membrane to generate a red line in the test line region (T). Presence of this red line indicates a positive result, while its absence indicates a negative result. Regardless of the presence of a test line, as the mixture continues to migrate across the membrane to the immobilized control line region, a red line at the control line region will always appear. The presence of this red line serves as verification for sufficient sample volume and proper flow and as a control for the reagents.

## PRECAUTIONS

- For research use only. Not for use in diagnostic procedures.
- The AMD has only been tested with human specimens.
- Do not use after expiration date.
- Handle all samples and kits used as if they contain infectious agents. Observe established precautions against microbiological hazards while performing all procedures and follow the standard procedures for proper disposal of blood and other samples and used kits.
- Wear protective clothing, eye protection and disposable gloves while performing the assay. Wash hands thoroughly when finished.
- Avoid all contact between hands and eyes or mucous membranes during testing.
- Do not eat, drink or smoke in the area where the samples and kits are handled.
- Chase Buffer contains a preservative; avoid all possible contact with skin and mucous membranes.

## STORAGE

The sealed pouch or vial containing the test strip and the bottles containing the kit buffers are designed to be stored at room temperature (20°C-28°C) for the duration of their shelf life. Exposure to temperatures over 30°C can impact the performance of the test and should be minimized. The strips should not be frozen. The test should be used within 30 minutes after removal from the pouch or vial to minimize exposure to humidity.

## SAMPLE COLLECTION-BLOOD

- Human whole blood (including finger pricked blood) and serum can be tested with this test strip.
- Use finger pricked blood, or collect blood in presence of anti-coagulants citrate, EDTA, or heparin.
- Test should be performed as soon as possible after sample collection. Do not leave blood at room temperature for prolonged periods. Whole blood can be refrigerated at 2-8°C up to 3 days.
- Bring samples to room temperature prior to testing.
- If samples are to be shipped, they should be packed in compliance with Federal Regulations covering transportation of infectious agents.
- See below for suggestions for other sample types.

## KIT CONTENTS

Active Melioidosis *Detect*™ test strip's membrane is pre-coated with a mouse monoclonal antibody on the test line region and a control antigen on the control line region.

The kit contains the following:

1. Twenty-five (25) individually pouched test strips or twenty-five (25) test strips in a vial with desiccant in the cap.
2. One (1) vial of Chase Buffer Type A solution.
3. One (1) vial of Lysis buffer (for non-blood related sample types).

Materials needed, but not provided:

1. Capillary tube capable of pipetting 35µl whole blood and/or serum
2. Single well buffer cup
3. Multichannel pipet for delivering 20, 100 or 150µl sample (for other sample types)
4. Eppendorf tubes (for other sample types)

## TEST PROCEDURE-BLOOD

1. Remove the Active Melioidosis *Detect*™ test strip from the foil pouch or vial.
2. Add 35 µl of whole blood, serum or plasma to the test strip and onto the colloidal gold conjugate coated area beneath the sample arrow tape (↓). For other sample types see below.
3. Transfer the test strip into a buffer well that has been pre-loaded with 3 drops of Chase buffer (untaped end of the strip facing downward).
4. Results MUST be read after *FIFTEEN (15)* minutes.

## TEST PROCEDURE-OTHER SAMPLE TYPES

The Active Melioidosis *Detect*™ has been used to test other sample types than whole blood, serum or plasma. These include testing bacterial colonies and cultures, sputum, pus and urine. Below are some suggestions for sample preparation for testing in the rapid test.

**Sputum**

**If sample is chunky/thick**

1. Add 150µl (3-4 drops) of lysis buffer to 20µl of sputum and mixed well by vortex mixer.
2. Add 20µl of sputum/lysis buffer to test strip followed by 150µl (3-4 drops) of Chase buffer.

**If sputum is thin**

1. Add 100µl (2-3 drops) of lysis buffer to 50µl of sputum and mixed well by vortex mixer.
2. Add 20µl of sputum/lysis buffer to test strip followed by 150µl (3-4 drops) of Chase buffer.

**Pus**

1. Add 100µl (2-3 drops) of lysis buffer to 20µl of pus and mixed well by vortex mixer.
2. Add 20µl of pus/lysis buffer to test strip followed by 150µl (3-4 drops) of Chase buffer.

**Urine**

**From Concentrate**

1. Spin down maximum of 10ml of urine at 3000-4000 rpm for 10 minutes.
2. Pour off supernatant and resuspend pellet in 50µl (1-2 drops) lysis buffer.
3. Add 20µl of urine/lysis buffer to test strip followed by 150µl (3-4 drops) of Chase buffer.

**Straight**

1. Add 50µl urine to 100µl (2-3 drops) Chase buffer.
2. Apply to test strip.

**Cultures**

**From culture Bottles**

1. Spin down maximum of 10ml of culture at 3000-4000 rpm (~ 3,200 x g) for 10 minutes.
2. Pour off supernatant and resuspend pellet in 50µl (1-2 drops) lysis buffer.
3. Add 20µl of culture/lysis buffer to test strip followed by 150µl (3-4 drops) of Chase buffer.

**From Bacterial isolates**

1. Pick a single colony and resuspend in 2 drops of lysis buffer and mix in an eppendorf tube.
2. Transfer bacterial suspension to test strip and immediately add 150µl (3-4 drops) of Chase Buffer.

**All above assays are read after 15 minutes.**

## INTERPRETATION OF RESULTS

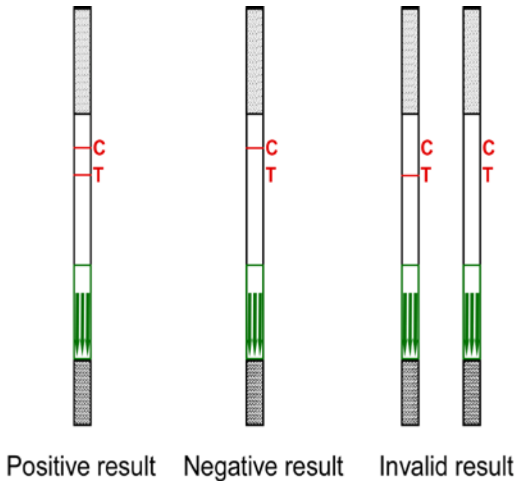


*Figure 1: Schematic representation of AMD reactivity*

**A Positive Result**

The test is positive when a control line (C) and test line (T) appears in the test area as shown in Figure 1A. A positive result indicates that the Active Melioidosis *Detect*™ dipstick detected capsular polysaccharide (CPS) antigen from *B. pseudomallei* in the sample. A faint line is considered a positive result. As a guide for interpretation, the red color in the test region (T) will vary depending on the concentration/affinity of antigens present.

**A Negative Result**

The test is negative when only the control line appears (Figure 1B). A negative result indicates that the Active Melioidosis *Detect*™ dipstick did not detect capsular polysaccharide (CPS) antigen of *B. pseudomallei.*

**An Invalid Result**

The test is invalid if no control line appears, regardless of a test line (Figure 1C). The test should be repeated with a fresh sample, if possible.

**Expected Value:**

Performance of Active Melioidosis *Detect* ™ has not been formally established. Therefore, sensitivity and specificity of the test cannot be claimed.

## LIMITATIONS

1. Do not use serum samples containing any glycerol or other viscous materials. This will decrease the sensitivity of the assay.

## REFERENCES


1. Nuti DE, Crump RB, Dwi Handayani F, Chantratita N, Peacock SJ, Bowen R, et al. Identification of circulating bacterial antigens by in vivo microbial antigen discovery. mBio. 2011;2(4)
2. Wiersinga WJ, Currie BJ, Peacock SJ. Melioidosis. The New England Journal of Medicine. 2012;367(11):1035-44.
3. Limmathurotsakul D, Wongratanacheewin S, Teerawattanasook N, Wongsuvan G, Chaisuksant S, Chetchotisakd P, et al. Increasing incidence of human melioidosis in Northeast Thailand. Am J Trop Med Hyg.82(6):1113-7.
4. Currie, B. J., L. Ward, et al. (2010). "The epidemiology and clinical spectrum of melioidosis: 540 cases from the 20 year Darwin prospective study." PLoS Negl Trop Dis **4**(11): e900.
5. Chaowagul W, Suputtamongkol Y, Dance DA, Rajchanuvong A, Pattara-arechachai J, White NJ. Relapse in melioidosis: incidence and risk factors. J Infect Dis. 1993;168(5):1181-5.
6. Warawa J, Woods DE. Melioidosis vaccines. Expert Rev Vaccines. 2002;1(4):477-82.

7. Inglis TJ, Merritt A, Chidlow G, Aravena-Roman M, Harnett G. Comparison of diagnostic laboratory methods for identification of *Burkholderia pseudomallei*. J Clin Microbiol. 2005;43(5):2201-6.
8 Wongsuvan G, Limmathurotsakul D, Wannapasni S, Chierakul W, Teerawattanasook N, Wuthiekanun V. Lack of correlation of *Burkholderia pseudomallei* quantities in blood, urine, sputum and pus. Southeast Asian J Trop Med Public Health. 2009;40(4):781-4.
9. Wuthiekanun V, Limmathurotsakul D, Wongsuvan G, Chierakul W, Teerawattanasook N, Teparrukkul P, et al. Quantitation of *B. pseudomallei* in clinical samples. Am J Trop Med Hyg. 2007;77(5):812-3.
10. Wuthiekanun V, Dance DA, Wattanagoon Y, Supputtamongkol Y, Chaowagul W, White NJ. The use of selective media for the isolation of *Pseudomonas pseudomallei* in clinical practice. J Med Microbiol. 1990;33(2):121-6.

InBios

InBios International, Inc.
562 1st Ave. South, Suite 600
Seattle, WA 98104 USA
Toll Free USA- 1-866-INBIOS1
206-344-5821 (International)
www.inbios.com

Insert Part No. 900137.01
Effective Date: 11/19/13
REF Catalog No. AMD-RD
